# 实时荧光定量PCR检测人肺腺癌细胞Anip973、Anip973/NVB中hTERT mRNA的表达

**DOI:** 10.3779/j.issn.1009-3419.2010.04.05

**Published:** 2010-04-20

**Authors:** 公琰 陈, 淑英 郭, 菁菁 柳

**Affiliations:** 1 150040 哈尔滨，哈尔滨医科大学附属肿瘤医院内一科 Department of Medical Oncology, Tumor Hospital of Harbin Medical University, Harbin 150040, China; 2 518055 深圳，广东省深圳市西丽人民医院 Department of Medical Oncology, Xili Hospital of Shenzhen, Shenzhen 518055, China; 3 130021 长春，吉林省肿瘤医院内一科 Department of Medical Oncology, Tumor Hospital of Jilin Province, Changchun 130021, China

**Keywords:** 肺腺癌, hTERT, 长春瑞滨, 实时荧光定量RT-PCR, 多药耐药, Lung neoplasms, hTERT, Navelbine, Real-time fluorescence quantitative RT-PCR, Multi-drug resistance

## Abstract

**背景与目的:**

端粒酶逆转录酶为端粒酶的催化亚单位，其活性与细胞对化疗药物的敏感性密切相关。本实验研究人肺腺癌Anip973/NVB耐药细胞株与亲代细胞Anip973细胞株端粒酶逆转录酶（human telomerase reverse transcriptase, hTERT）的mRNA表达及差异，探讨端粒酶与耐药的相关性。

**方法:**

采用实时荧光定量RT-PCR法检测人肺腺癌Anip973/NVB耐药细胞与Anip973亲代细胞经NVB处理前后hTERT mRNA的表达。

**结果:**

对照组Anip973、Anip973/NVB细胞的hTERT mRNA表达无统计学差异；NVB处理组Anip973细胞hTERT mRNA表达明显降低（*P* < 0.01），而Anip973/NVB细胞hTERT mRNA表达的降低与对照组比较无统计学差异（*P* > 0.05），NVB处理组Anip973细胞hTERT mRNA表达降低的程度明显高于Anip973/NVB细胞（*P* < 0.01）。

**结论:**

端粒酶与肺腺癌耐药细胞的多药耐药密切相关，端粒酶有可能作为逆转耐药的新靶点。

## Abstract (en)

**Background and objective:**

Human telomerase reverse transcriptase is the catalytic subunit of telomerase, and its activity is correlated with cell's sensitivity to chemotherapy. The aim of this study is to investigate the differential expression of human telomerase reverse transcriptase (hTERT) mRNA in human lung adenocarcinoma cell line Anip973 and Anip973/NVB, and to observe the correlation between hTERT mRNA and drug-resistance.

**Methods:**

The real-time fluorescence quantitative RT-PCR was used to detect the change of hTERT mRNA in human lung adenocarcinoma drug-resistant cell Anip973/NVB and parental cell Anip973 treated by NVB.

**Results:**

In the control group, the expression of hTERT mRNA showed no significant difference between drug-resistant cell Anip973/NVB and parental cell Anip973. After been treated by NVB, the expression of hTERT mRNA in parental cell was significantly decreased (*P* < 0.01), and drug-resistant cell Anip973/ NVB had no evidently variant (*P* > 0.05). The down-regulated hTERT mRNA in Anip973 cell was higher than that in Anip973/ NVB cell.

**Conclusion:**

Telomerase correlates with the drug-resistant cell A973/NVB, and telomerase may be a new target for multi-drug resistant inversion.

肺癌是全世界常见的恶性肿瘤，是人类恶性肿瘤死亡的主要原因。由于三分之二的肺癌病人就诊时已属晚期，失去了手术机会，而化疗是中晚期肺癌治疗最重要的有效手段。然而化疗过程中肿瘤细胞产生耐药是化疗失败的主要原因之一，也是治疗肿瘤的主要难题，如何克服肿瘤细胞多药耐药是目前临床治疗所面临的巨大挑战。端粒酶是一种细胞核糖核蛋白酶，端粒酶RNA片断可作为模板将端粒DNA合成到染色体末端^[1]^。端粒酶与肿瘤细胞的增殖和永生化密切相关，端粒酶的激活可以使端粒长度不断延长，从而使细胞无限增殖形成肿瘤。端粒酶逆转录酶（human telomerase reverse transcriptase, hTERT）为端粒酶的催化亚单位，是端粒酶活化的关键成分，与端粒酶活性密切相关。相关研究^[2, 3]^表明端粒酶活性与细胞对化疗药物的敏感性密切相关，端粒酶活性高的病人化疗效果差，说明端粒酶有可能参与了多药耐药机制。本研究采用实时荧光定量RT-PCR法检测经长春瑞滨（Navelbine, NVB）处理前后人肺腺癌耐药细胞Anip973/NVB与亲代细胞Anip973的hTERT mRNA表达水平，探讨端粒酶是否参与肿瘤的耐药机制。

## 1 材料与方法

### 1.1 细胞株

人肺腺癌细胞株Anip973由黑龙江省肿瘤研究所惠赠，人肺腺癌耐药细胞株Anip973/NVB由作者前期诱导建立^[4]^，NVB终浓度为2.0 μg/mL。细胞常规培养于含10%胎牛血清、100 U/mL青霉素、100 μg/mL链霉素的RPMI-1640培养基中，于37 ℃、5%CO_2_的饱和湿度孵箱培养。每2天-3天传代一次，每日观察细胞形态，取对数生长细胞用于实验。Anip973细胞和Anip973/NVB细胞均加入浓度为2.0 μg/mL的NVB，培养48 h后进行实验。

### 1.2 药物与试剂

NVB购自法国皮尔·法伯药物研制公司；二甲基四氮唑蓝（MTT）购自Sigma；顺铂（CDDP）购自科鼎医疗有限公司；表阿霉素（EPI）购自浙江海工药业股份有限公司；健择（GEM）购自礼来公司；伊立替康（CPT-11）购自辉瑞公司；足叶乙甙（VP-16）丽珠集团利民制药厂；RNA提取试剂盒购自上海生工生物工程有限公司；RT-PCR试剂盒购自广州达晖生物技术有限公司；引物、Taqman探针由广州达晖生物技术有限公司合成。

### 1.3 MTT法测定细胞抗药性

取对数生长期的Anip973细胞和Anip973/NVB细胞，以1×10^4^/mL的浓度接种于96孔培养板，每孔180 μL，37 ℃、5%CO_2_条件下培养4 h，分组加药，每个浓度设3个平行孔，处理组加入培养液稀释的不同浓度的各种化疗药物（长春瑞滨、顺铂、表阿霉素、健择、依立替康、足叶乙甙）各20 μL，阴性对照组加生理盐水，培养48 h后，每孔加入20 μL的MTT溶液，继续培养4 h后，甩板去除培养液后每孔加入二甲基亚砜150 μL，振荡至沉淀完全溶解，在全自动酶标仪上选择波长490 nm测定吸光值（*A*值），根据*A*值计算各药物的半数抑制率（IC_50_）。根据各组细胞IC_50_与敏感细胞IC_50_的比值计算耐药指数（resistance index, RI）。实验重复3次，取平均值。

### 1.4 引物与探针

引物、Taqman探针由广州达晖生物技术有限公司合成，见表 1。

**1:** 引物和Taqman探针序列 The sequences of primers and Taqman probes

Gene and oligo-nucleotide		Sequence
TERT-extra	Forward Primer	AGAGCACCGTCTGCGTGAGG
	Reverse Primer	AACTTGCTCCAGACACTCTT
TERT-intra	Forward Primer	TTCCTGCACTGGCTGATGAG
	Reverse Primer	GAAACGTGGTCTCCGTGACA
TaqMan Probe		TGTACGTCGTCGAGCTGCTCAGGTCT
GAPDH	Forward Primer	GGTGAAGGTCGGAGTCAACGG
	Reverse Primer	CCTGGAAGATGGTGATGGGATT

### 1.5 细胞总RNA提取

采用UNIQ-10柱总RNA提取试剂盒提取RNA，实验步骤按说明书进行。使用紫外分光光度仪来检测提取总RNA的含量及纯度。要求*A*_260_/*A*_280_的比值均在1.8-2.0之间，说明RNA纯度较高，未被蛋白质、酚污染，完全可以满足RT-PCR需要。

### 1.6 cDNA的合成

反应体系20 μL。包括5×逆转录buffer [50 mmol Tris-HCl（pH8.0）, 50 mmol KCl, 4 mmol MgCl_2_, 10 mmol DTT] 4 μL，上游外引物（10 pmol/μL）0.4 μL，下游外引物（10 pmol/μL）0.4μL，dNTPs（10 mmol/μL）0.2 μL，MMLV（200 U/μL）1 μL，DEPC水9 μL，RNA模板5 μL。反应条件为37 ℃、60 min，95 ℃、3 min。

### 1.7 荧光定量PCR反应

反应体系50 μL。包括5×PCR buffer [10 mmol Tris-HCl（pH8.0）, KCl 50 mmol, MgCl_2_ 2 mmol]10 μL，上游内引物F（10 pmol/µL）1 μL，下游内引物R（10 pmol/µL）1 μL，dNTPs（10 mmol）1 μL，cDNA产物5 μL，ddH_2_O 32 μL。反应条件为93 ℃、2 min，93 ℃、45 s，55 ℃、1 min，共40个循环。

### 1.8 标准曲线的绘制

标准品质粒的构建由广州达晖生物技术有限公司完成，作为制备标准曲线的模板，与待测样品同时进行PCR扩增，绘制标准曲线。用2^-ΔΔCt^法计算各个样品的hTERT表达量，其中ΔCt =检测样品Ct值-内参Ct值；ΔΔCt=阳性待测组样品ΔCt-正常样品组ΔCt。

### 1.9 统计学方法

应用SPSS 13.0软件对实验数据进行统计学分析，两两比较用*t*检验，以*P* < 0.05为有统计学差异。

## 2 结果

### 2.1 耐药谱分析

MTT法检测耐药细胞与亲代细胞对各种抗癌药的细胞毒作用，结果表明Anip973/NVB对NVB的耐药指数为27.43，并对伊立替康、顺铂、足叶乙甙、表阿霉素等未接触过的抗肿瘤机制不同的抗癌药物也产生交叉耐药（*P* < 0.05），见表 2，表明其耐药性稳定。

**2:** Anip973和Anip973/NVB细胞药物敏感性对比 The contrast of drug sensitivity between Anip973 and Anip973/NVB cell

Drugs	IC_50_ (A973/NVB)(μg/mL)	IC_50_(A973)(μg/mL)	Resistance index
Navelbine	10.38	0.38	27.43
Irinotecan	853.42	44.31	19.26
Cisplatin	0.48	0.04	12.49
Etoposide	83.24	10.45	7.96
Epirubicin	46.75	10.95	4.27
Gemcitabine	307.53	287.41	1.07

### 2.2 总RNA的OD值及浓度

RNA样品*A*_260_/*A*_280_的比值均在1.8-2.0之间，说明RNA纯度较高，未被蛋白质、酚污染，完全可以满足RT-PCR需要。RNA的浓度：Anip973细胞为1.008 μg/μL，Anip973/NVB细胞为0.912 μg/μL。

### 2.3 hTERT mRNA标准品荧光曲线与标准曲线

图 1为梯度稀释的hTERT mRNA标准品的动态扩增曲线，根据动态扩增曲线建立标准曲线，以模板稀释倍数的对数值为横坐标，循环阈值（cyle threshold, Ct）值为纵坐标，表明在1×10^5^-1×10^8^范围内以10倍梯度稀释这样一个大的跨度范围内，DNA的拷贝数与Ct之间存在良好的线性关系，统计学分析相关系数R_2_ > 0.99，线性关系极好。图 2显示不同起始浓度模板进入PCR指数增长期的起始点即不同，拷贝数越大Ct值越小。不同浓度的模板进入PCR平台期后，PCR产物差异也比较大且不成比例。不同浓度标准品的Ct值与该标准品浓度的对数存在线性关系，起始浓度越高，Ct值就越小。

**1:**
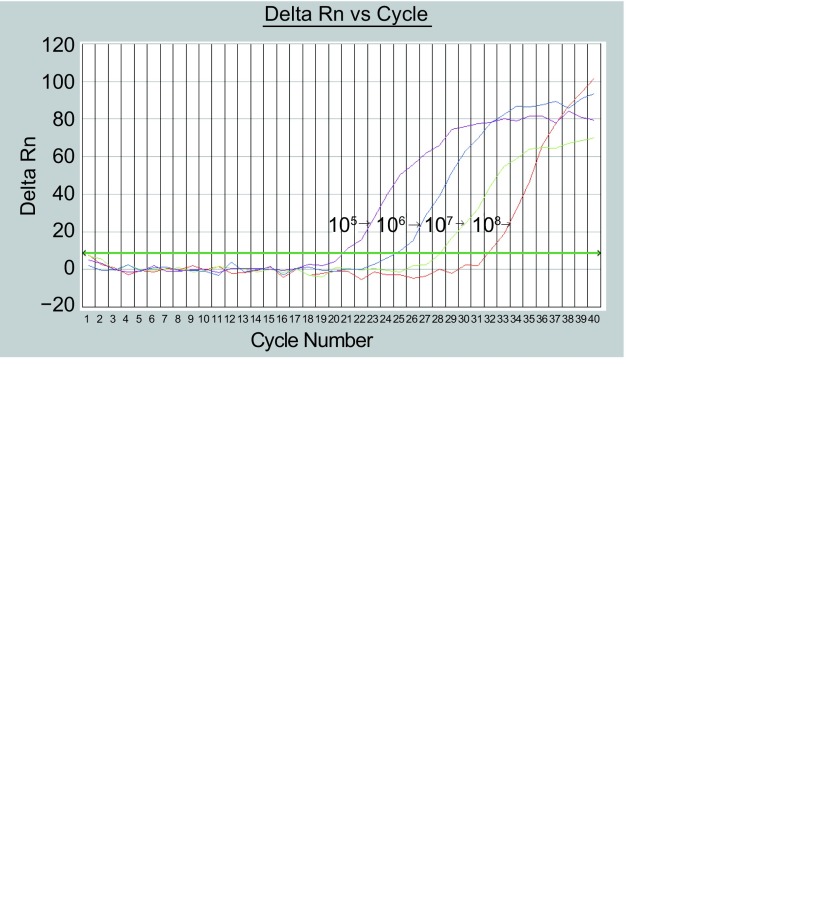
不同模板浓度下hTERT实时PCR荧光曲线 Real-time flourescence curves of hTERT in different template quantity From left to right the concentrations of templates are: 1×10^5^, 1×10^6^, 1×10^7^, 1×10^8^.

**2:**
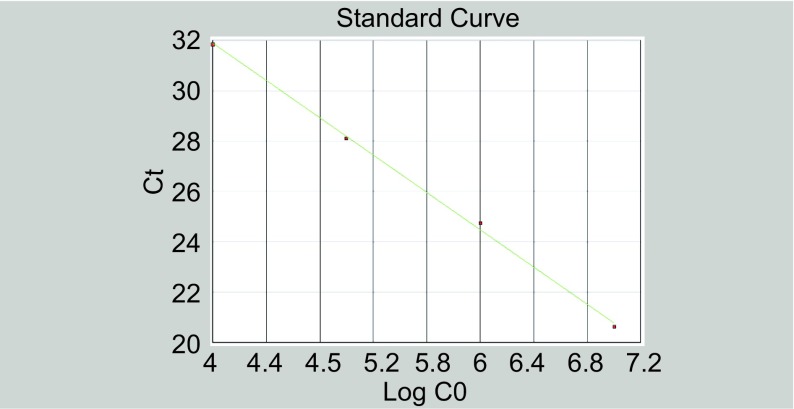
hTERT的实时PCR标准曲线 Standard real-time PCR curve of hTERT

### 2.4 NVB对Anip973和Anip973/NVB细胞hTERT mRNA表达的影响

2.0 μg/mL NVB处理前后亲代细胞与耐药细胞的hTERT mRNA表达见表 2，反应后的荧光曲线见图 3。对照组Anip973和Anip973/NVB细胞hTERT mRNA表达差异无统计学意义（*P* > 0.05）；NVB处理组Anip973细胞hTERT mRNA表达显著降低，与对照组Anip973细胞比较有统计学差异（*P* < 0.01）；NVB处理组Anip973/NVB细胞hTERT mRNA表达亦有降低，但与对照组Anip973/NVB细胞比较差异无统计学意义（*P* > 0.05）；在NVB处理组，Anip973细胞hTERT mRNA表达的降低程度明显高于Anip973/NVB细胞（*P* < 0.01），有统计学差异（表 3）。

**3:**
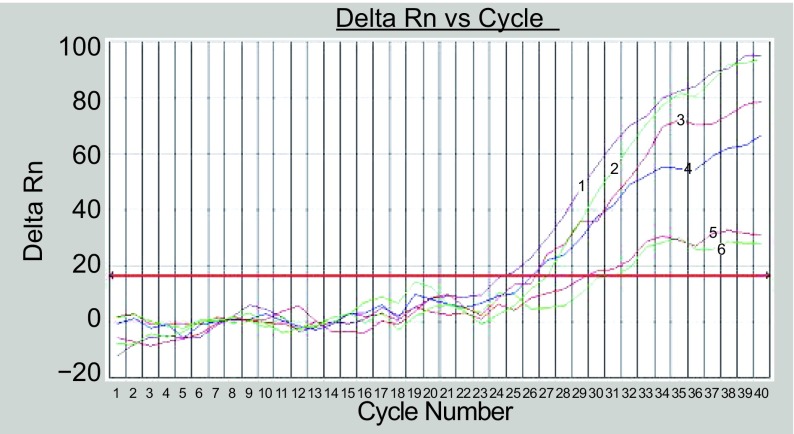
Anip973/NVB和Anip973细胞hTERT的实时PCR荧光曲线 Real-time PCR flourescence curves of Anip973/NVB and Anip973 1, 2, 3: Anip973 cell; 4, 5, 6: Anip973/NVB cell.

**3:** Anip973和Anip973/NVB细胞hTERT mRNA的表达 Expression of hTERT mRNA in Anip973 and Anip973/NVB cell

Group	Expression of hTERT mRNA	
	Anip973	Anip973/NVB
Control group	5.59±0.62	7.40±0.65^△^
Experimental group	1.14±0.11^*^	6.21±0.42^#^
^*^: Anip973 experimental group *vs* Anip973 control group, Anip973 experimental group *vs* Anip973/NVB experimental group, *P* < 0.01; ^△^: Anip973/NVB control group *vs* Anip973 control group, *P* > 0.05; ^#^: Anip973 experimental group *vs* Anip973/NVB control group, *P* > 0.05.		

## 3 讨论

肿瘤细胞的多药耐药（multidrug resistance, MDR）是导致肿瘤化疗失败的主要原因之一。端粒酶是一种核糖核蛋白复合物，hTERT是端粒酶活性调控的限速因子，*hTERT*基因的表达与端粒酶活性表达一致。在肿瘤发生过程中，端粒酶的再激活可能参与细胞癌变过程。有研究^[2, 5]^表明抑制端粒酶活性可提高细胞对化疗药物的敏感性，化疗药物也可降低肿瘤细胞的端粒酶活性，hTERT mRNA可能与MDR1、MRP-mRNA及C-myc相关^[6]^，提示端粒酶可能参与了肿瘤细胞的耐药机制。

本实验以人肺腺癌Anip973、Anip973/NVB细胞为研究对象。Anip973/NVB细胞是通过逐渐增加剂量法诱导而成，是稳定的肺癌获得性多药耐药细胞系^[4]^，用MTT法进行耐药谱分析，Anip973、Anip973/NVB细胞48 h的IC_50_值分别为0.38 μg/mL、10.38 μg/mL，RI为27.43，对伊立替康等四种药物也产生了交叉耐药，说明其耐药性稳定。用实时荧光定量RT-PCR法检测了Anip973、Anip973/ NVB细胞hTERT mRNA的表达情况，在模板稀释1 000倍时，hTERT标准品表现为阳性扩增，呈明显的S型，其灵敏度达到了极限，可以检测到微量的基因，这是传统的PCR难以做到的，说明实时PCR技术有较高的灵敏度。本研究结果表明Anip973、Anip973/NVB细胞均有端粒酶hTERT mRNA表达，对照组亲代细胞和耐药细胞的hTERT mRNA表达无统计学差异（*P* > 0.05），NVB处理组Anip973细胞hTERT mRNA表达明显降低（*P* < 0.01），而Anip973/NVB细胞hTERT mRNA表达虽有下调趋势，但与对照组耐药细胞比较差异无统计学意义（*P* > 0.05），同时NVB处理组Anip973/NVB细胞hTERT mRNA表达明显高于Anip973细胞（*P* < 0.01），差异有统计学意义，说明在化疗药物长期刺激下，端粒酶的表达活性更加稳定且能保持持续活化，细胞自身修复能力明显增强，使得耐药细胞对化疗药物具有更加良好的抵抗性和适应性。由此可见，端粒酶与肿瘤细胞多药耐药具有一定的相关性，这与相关文献报道相似^[7, 8]^。

端粒酶在维持长度、保持染色体稳定性方面有重要作用，在绝大多数肿瘤细胞中都有端粒酶高表达，正常细胞中端粒酶不表达或低表达。在正常组织和肿瘤组织中端粒酶表达、端粒长度和细胞动力学的差别显示出以端粒酶作为治疗靶点会相对安全，抑制端粒酶能有效提高耐药细胞对化疗药物的敏感性^[9]^，针对端粒酶的反义治疗有利于逆转肿瘤细胞对化疗药物的耐药性^[10, 11]^，提示端粒酶抑制剂可治疗肿瘤并有可能逆转肿瘤细胞耐药。本实验利用实时荧光定量RT-PCR法检测hTERT mRNA表达，证实端粒酶可能参与了人肺腺癌Anip973/ NVB细胞的MDR，提示可将端粒酶作为逆转耐药的新靶点，至于如何利用端粒酶抑制剂逆转耐药及如何将其应用于临床仍需在今后的研究中进一步探索。
